# Differences in muscle satellite cell dynamics during muscle hypertrophy and regeneration

**DOI:** 10.1186/s13395-022-00300-0

**Published:** 2022-07-06

**Authors:** So-ichiro Fukada, Tatsuyoshi Higashimoto, Akihiro Kaneshige

**Affiliations:** 1 Project for Muscle Stem Cell Biology, Graduate School of Pharmaceutical Sciences, Osaka University, 1-6 Yamada-oka, Suita, Osaka, 565-0871 Japan; 2 Biological/Pharmacological Research Laboratories, Central Pharmaceutical Research Institute, Japan Tobacco Inc., 1-1 Murasaki-cho, Takatsuki, Osaka, 569-1125 Japan

**Keywords:** Muscle regeneration, Hypertrophy, Muscle satellite cells, Myonuclei, Differentiation

## Abstract

Skeletal muscle homeostasis and function are ensured by orchestrated cellular interactions among several types of cells. A noticeable aspect of skeletal muscle biology is the drastic cell–cell communication changes that occur in multiple scenarios. The process of recovering from an injury, which is known as regeneration, has been relatively well investigated. However, the cellular interplay that occurs in response to mechanical loading, such as during resistance training, is poorly understood compared to regeneration. During muscle regeneration, muscle satellite cells (MuSCs) rebuild multinuclear myofibers through a stepwise process of proliferation, differentiation, fusion, and maturation, whereas during mechanical loading-dependent muscle hypertrophy, MuSCs do not undergo such stepwise processes (except in rare injuries) because the nuclei of MuSCs become directly incorporated into the mature myonuclei. In this review, six specific examples of such differences in MuSC dynamics between regeneration and hypertrophy processes are discussed.

## Background

**Fig. 1:**
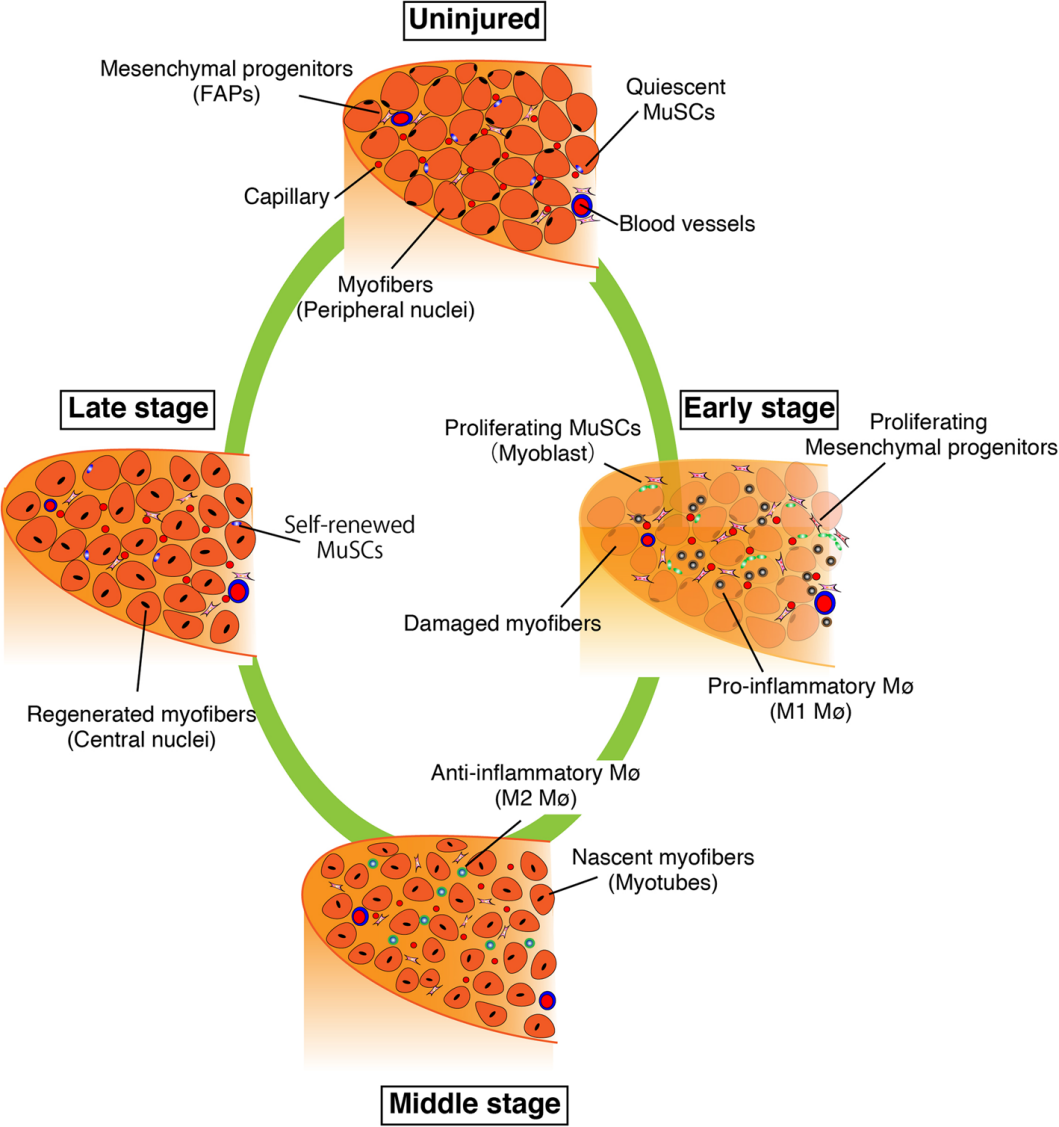
Process of skeletal muscle regeneration. When myofibers are damaged or dead, their debris is removed by inflammatory macrophages (M1 Mø). Using spaces and factors derived from macrophages and mesenchymal progenitors (FAPs), muscle satellite cells (MuSCs) actively proliferate (early stage). In the middle stage of regeneration, anti-inflammatory macrophages (M2 Mø) support the regulation of myogenic differentiation and nascent myofibers (myotubes), which grow to mature myofibers (late stage)

**Fig. 2:**
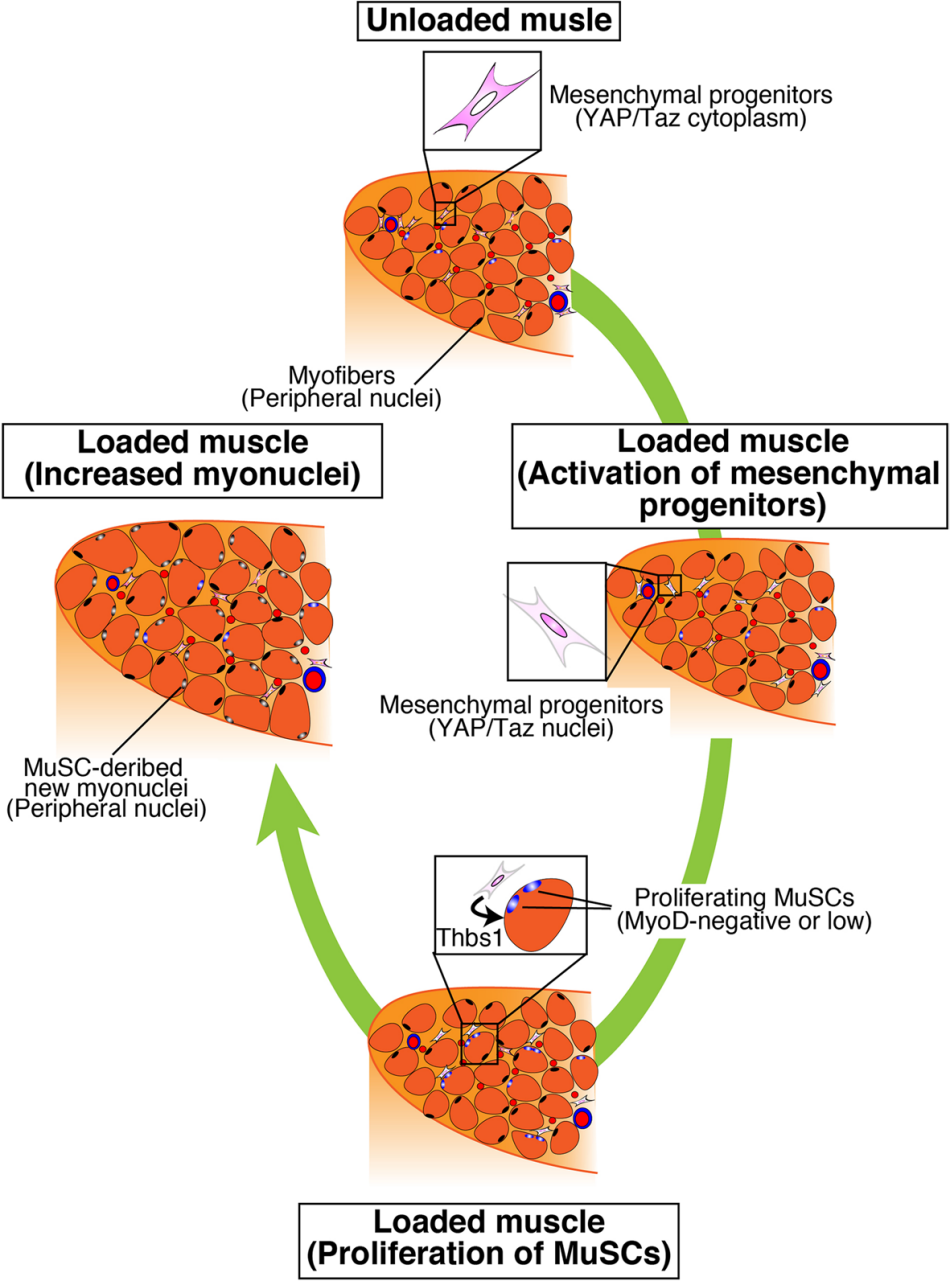
Process of mechanical-loaded muscle hypertrophy. In unloaded muscles, Yap/Taz is distributed in the cytoplasm of mesenchymal progenitors. Mechanical loading induces nuclear localization of Yap/Taz in mesenchymal progenitors, and MuSCs subsequently proliferate beneath the basal lamina by the mesenchymal progenitor-derived factor thrombospondin-1 (Thbs1). Proliferated MuSCs fuse with myofibers, which leads to an increased number of myonuclei. Notably, the new myonuclei are located in the peripheral position of myofibers

Skeletal muscle is a dynamic tissue that presents excellent regenerative ability and plasticity in response to external and internal changes, and both processes rely on myogenic-committed cells that reside in skeletal muscle, which are known as muscle satellite cells (MuSCs) [1–4]. MuSCs remain in a quiescent state under steady conditions [5] but start proliferating in response to damage or skeletal muscle loading. MuSC behavior is affected by multiple cell types, including myofibers, immune cells, and interstitial cells, including endothelial cells and mesenchymal progenitors (FAPs: fibro/adipogenic progenitors) (Figs. 1 and 2) [6–11]. Compared to the process of muscle regeneration, the mechanism regulating MuSC dynamics under muscle loading (such as a resistance training) has not been well investigated. However, recent studies have analyzed the mechanisms underlying MuSC proliferation and cell–cell communication in loaded muscles [9, 10, 12, 13]. We briefly summarize the process of muscle regeneration and load-dependent muscle hypertrophy according to key factors underlying MuSC behaviors and discuss six differences in MuSC dynamics and cell–cell interactions between the regeneration and hypertrophy processes.

### Muscle regeneration

When a myofiber is damaged, MuSCs exit from the quiescent state and become activated and proliferate (Fig. 1). MuSC activation follows after the expression of myoblast determination protein 1 (MyoD) [14, 15]. Subsequently, genes that regulate the cell cycle are upregulated and the cells begin to proliferate. In vitro experiments have revealed that approximately 2 days are required for the first cell division of quiescent MuSCs [16]. The subsequent cell division rate, which is estimated to be 12 h in vitro, is considerably faster than the initial cell division rate [16]. In a cardiotoxin (CTX)-induced muscle injury model in C57BL/6 mice, day 3 after injury corresponds to the peak number of proliferating myoblasts (daughter cells of MuSCs) [17] while day 4 after injury corresponds to the abundant production of immature myofibers called myotubes, which are generated by myoblast–myoblast and myoblast–nascent myotube fusion (Fig. 1). Active myoblast-dependent myotube growth occurs from days 5–10 postinjury [17]. During this process, MuSCs present a self-renewal capacity that maintains the number of MuSCs and ensures their ability to repeatedly regenerate after future damages. Although the exact timing of MuSC self-renewal is unknown, studies have suggested that self-renewal determination occurs in the mid-regeneration period (approximately 4–5 days after injury) [17, 18]. In addition, Pawlikowski et al. reported that the majority of MuSC self-renewal occurs between days 5 and 7 postinjury since EdU-labeling experiments demonstrated that the last MuSC division occurred during regeneration in this period [19]. In mice with CTX-induced muscle injury, myofibers are rebuilt to their original size approximately 2–3 weeks after injury (Fig. 1).

For efficient MuSC proliferation and differentiation, inflammatory cells, mesenchymal progenitors, and basal lamina are required [7, 20–22]. The infiltration of neutrophils is observed within 12 h [23], although the primary infiltrating cells subsequently shift to macrophages, which clean up dead myofibers [24]. Consequently, the space for MuSC proliferation is ensured. In the early stages of muscle regeneration, inflammatory M1 macrophages are the main subset, and they are then replaced by anti-inflammatory M2 macrophages (Fig. 1) [25]. Both macrophages and mesenchymal progenitors contribute to MuSC proliferation [6, 7, 24]. Similar to MuSCs, the number of mesenchymal progenitors and macrophages reach their peak approximately 3 days after CTX injection and then return to their original number [26].

### Muscle hypertrophy

The crucial feature of overload-induced skeletal muscle hypertrophy is an increase in myofiber size, which requires two events: an increase in protein synthesis and then an increase in myofiber nuclei [27, 28]. The insulin-like growth factor 1 (IGF1)-Akt-mammalian target of rapamycin (mTOR) pathway is a well-known protein synthesis pathway. Akt also suppresses the Forkhead box O (Foxo) transcription factor, thereby inhibiting the ubiquitin–proteasomal and autophagic/lysosomal pathways [29, 30]. Other pathways, such as calcium signaling, have also been reported to activate mTOR [31], and these pathways were extensively summarized in our recent review [28].

An increased number of myonuclei by MuSCs is also required for efficient muscle hypertrophy (Fig. 2). Mice depleted of MuSCs did not exhibit increases in myonuclei [3, 32], indicating that myonuclei accretion, as well as myofiber generation, absolutely depends on MuSCs [1, 2]. The need for an increased number of myonuclei in muscle hypertrophy or MuSCs had been debated for two decades [3, 33, 34]. Although experimental conditions or methodologies may obscure the effect of increased myonuclei on the efficiency of muscle hypertrophy over relatively short-term (2–3 weeks) periods after surgical mechanical loading [9, 32], all recent studies have demonstrated that an increase in the myonuclei number is critical for long-term (>8 weeks) muscle hypertrophy [9, 28, 35]. Moreover, the increased myonuclear number and protein synthesis during muscle hypertrophy are coordinated because the disruption of new myonuclear accretion in overloaded muscle results in reduced Akt activation and downstream signaling [36]. Collectively, the data indicate that myonuclear accretion is required for sustained functional growth.

In our tenotomy-induced overloaded model, MuSCs started to express Ki67 at 2 days and substantial MuSC proliferation was observed approximately 4 days after surgery. Although the number was small, new MuSC-derived myonuclei were detected 4 days after tenotomy, and the number of MuSC-derived myonuclei gradually increased at least 2 weeks after surgery [12]. In loaded muscle, MuSC proliferation and differentiation seemed to occur concurrently (Fig. 2) [12, 37].

Compared with skeletal muscle regeneration processes, studies on cell–cell interactions during loading-dependent muscle hypertrophy are limited because the role of MuSCs is linked to their regenerative capacity, even in loaded muscle [37]. Undeniably, exercise is a widely accepted model of skeletal muscle loading that damages myofibers, particularly in rodent models [38]. Equivalent or similar experimental models have also been used to study the signaling pathways involved in the hypertrophy of living myofibers. Notably, the term “damaged myofiber” is based on physiological events (damage to the myofibril structures and damage to the myofiber sarcolemma with or without myofiber death; thus, in this study “damage” refers to “damage causing myofiber death”) [37]. In our surgical overload model, the areas of dead myofibers were rare, many living myofibers could be easily isolated, and MuSC proliferation on myofibers was observed from the loaded muscle, indicating that MuSC behaviors in the loaded muscles are regulated by different pathways compared with the regeneration process [12]. Collectively, these results led us to speculate on the differences in MuSC dynamics, cell–cell interactions, and their mechanism between muscle regeneration and hypertrophy processes (Figs. 1 and 2). Six differences are discussed below based on a comparison of the processes of muscle regeneration and hypertrophy among recent and other published studies.

### Different activation and proliferation factors

Myofibers provide a specialized environment for MuSCs to sustain their undifferentiated and quiescent state. Notably, simply detaching MuSCs from myofibers may cause their activation and associated gene expression changes. Machado et al. demonstrated that the expression of early response genes, such as *Jun*, *Egr1*, and *Fosb*, is quickly upregulated in isolated MuSCs during cell preparation compared to that of *bona fide* quiescent MuSCs [39]. Therefore, during muscle regeneration, the loss of myofibers or factors secreted from damaged myofibers induce the activation and proliferation of MuSCs. For example, the secretion of tenascin-C [40] or GAPDH [41] from dead myofibers has been shown to induce the activation and proliferation of MuSCs. Several macrophage-derived factors (TWEAK, GFD3, GDF15, and IGF1) have been identified as regulators of MuSC proliferation and differentiation [42–44]. Mesenchymal progenitors also express factors that promote MuSC proliferation [6], including a matricellular protein named WISP1 (WNT1 inducible signaling pathway protein 1, also known as Ccn4), whose downregulation during aging is involved in the reduced proliferation of MuSCs in aged mice [45]. In addition, mesenchymal progenitors are critical for the infiltration of hematopoietic cells, including macrophages, into damaged muscles [6]. Collectively, the interplay among mesenchymal progenitors, macrophages, and MuSCs is critical for the successful progression of muscle regeneration.

On the other hand, the environment of MuSCs in overload-dependent muscle hypertrophy is not significantly altered because it does not accompany myofiber loss (Fig. 2) [37, 46]. Several mechanisms may be responsible for inducing the activation and proliferation of MuSCs:

1. Edema observed in early overloaded muscles [47]
2. Direct mechanical forces acting on MuSCs
3. Factors leaked from myofiber wounds that are not involved in cell death
4. Factors secreted from myofibers in a mechanical force-dependent manner

Additional factors may also be considered. Recently, in surgically overloaded plantaris muscles, we found that mesenchymal progenitors are critical for efficient muscle hypertrophy by regulating MuSC proliferation (Fig. 2) [9]. In this model, an initial, likely edema-induced, increase in muscle weight in mesenchymal progenitor-depleted mice was comparable to that observed in control mice. Meanwhile, the activation and proliferation of MuSCs were severely impaired by the loss of mesenchymal progenitors, suggesting that edema is unlikely to induce MuSC activation and proliferation [9]. In addition, the ability of MuSCs to directly sense mechanical forces should be similar in control and mesenchymal progenitor-deficient mice, making it unlikely that (1) and (2) alone would induce MuSC proliferation. Although strictly distinguishing (3) from (4) may be difficult, the ability of myofiber-derived factors to affect the proliferation and dynamics of MuSCs in loaded muscles has been clarified. Myofiber-derived IL-6 has been well investigated as an exercise-dependent factor that promotes MuSC proliferation [48, 49]. Succinate acid from exercised myofibers also affects MuSC gene signatures [50]. The relevance of mesenchymal progenitors and myofibers should be further investigated to reveal the entire mechanism that regulates MuSC activation and proliferation in loaded muscles.

We also found that mesenchymal progenitors secrete various growth factors in response to increased mechanical force via Yap1/Taz (Fig. 2), which are known as mechano-transducers [9]. In particular, we demonstrated that thrombospondin-1 (Thbs1), a member of the matricellular protein family derived from mesenchymal progenitors through Yap1/Taz, promotes MuSC proliferation by stimulating CD47 expressed on MuSCs in loaded muscle (Fig. 2) [9]. Mesenchymal progenitors actively proliferate in damaged muscles but show limited proliferation in overloaded muscles, suggesting that their dynamics differ between regenerating and hypertrophic muscles. Meanwhile, our RNA-seq data indicate that mesenchymal progenitors from loaded muscle also express WISP1 and chemokines that recruit inflammatory cells [9], suggesting that, in part, mesenchymal progenitors have common functions between regenerating and overloaded muscles. Further studies are required to reveal cell–cell communications, including the mesenchymal progenitor–myofiber or mesenchymal progenitor–macrophage axis.

### Differences in proliferation sites

MuSCs proliferate at different locations during muscle regeneration and hypertrophy. In regenerating muscle, macrophages clean up dead myofibers while the basal lamina that surrounds myofibers is retained. MuSCs proliferate beneath the retained basal lamina, called ghost myofiber [22]. Ghost fibers are essential for MuSCs to proliferate as a scaffold because muscle regeneration is delayed when ghost fibers are destroyed by ficin, which is a protease from fig trees [51]. In the middle of muscle regeneration (4–5 days after CTX injection), approximately 15% of Pax7-positive cells are located in the interstitial area, and doublecortin (the mutation is known to cause human lissencephaly) allows them to migrate into the basal lamina [17]. The characteristics of interstitial MuSCs, particularly their self-renewal ability, remain to be clarified.

Although MuSCs proliferate in the space between the basal lamina and sarcolemma during muscle hypertrophy [12], Pax7-positive cells have not been observed in the interstitial space. To examine whether MuSCs proliferate on living myofibers, we isolated and analyzed myofibers from overloaded muscles 4 days after tenotomy and succeeded in observing clusters of proliferating MuSCs on myofibers from overloaded muscle [12]. Myofibers easily die during the isolation process if they sustain any damage. Therefore, the clusters of MuSCs observed on immediately isolated myofibers provide proof that MuSCs proliferate on living and non-damaged myofibers in vivo.

### Differential expression of myogenic regulatory and Notch-related genes

As previously described, the expression of MyoD, a member of the myogenic regulatory factor family, is used to define MuSC activation. Compared to activated and proliferating MuSCs, the transcript level of *MyoD* is very low in quiescent MuSCs, in which canonical Notch signaling is higher. Of note, MyoD transcription partly occurs even in quiescent MuSCs, and the intron is retained to prevent the production of mature *MyoD* mRNA [52]. As *MyoD* repression by Notch signaling occurs at the transcriptional level, MyoD protein expression is repressed in MuSCs at both translational and transcriptional levels via Notch signaling and intron-retaining mechanisms. During MuSC isolation, the downregulation of Notch-related genes and *MyoD* intron-retained transcripts was observed [52, 53]. Considering that the loss of the Notch ligand Dll4 on myofibers leads to increased MyoD expression in quiescent MuSCs [54], these results also indicate that the induction of MyoD protein expression is an early event in MuSC activation by detachment from myofibers.

In overloaded muscles, MyoD is not expressed in proliferating MuSCs or is expressed at a low level that cannot be detected by antibodies [12]. Canonical Notch signaling is also a considerable mechanism for suppressing MyoD expression in proliferating MuSCs on loaded myofibers. In fact, representative and functional Notch signaling target genes in MuSCs, such as *HeyL* and *Col5a1* [55, 56], are persistently expressed in proliferating MuSCs in overloaded muscles [12]. Notably, the expression of *Hey1*, which is another representative Notch signaling target gene [55], was decreased in proliferating MuSCs. As the expression of *Hey1* is also present at high levels in quiescent MuSCs [57], the regulation of Notch signaling is expected to differ between quiescent and proliferating MuSCs in overloaded muscles.

### Differences in fusion partners

During muscle regeneration, proliferating myoblasts initially fuse with each other to form multinucleated cells called myotubes (Fig. 1). Subsequently, the nuclear number of myotubes is increased by the additional fusion of myoblasts. Several events are involved in the formation of myotubes and myofibers, including cell migration, recognition, adhesion, and fusion, and fusion-related molecules have been identified [58]. Among them, Myomaker and Myomixer (also known as Myomerger and Minion) [59–62] are necessary and sufficient cell fusion molecules because the expression of these two factors allows non-myogenic cells, like fibroblasts, to fuse and form multinucleated cells.

In contrast, MuSCs directly fuse with mature myofibers during muscle hypertrophy (Fig. 2). Thus, although the fusion partners are different, Myomaker expressed by MuSCs is also essential for fusion with myofibers [36]. However, whether the expression of Myomaker and Myomixer by myofiber is also necessary for the fusion of proliferating MuSCs and myofibers has not been clarified. Notably, a developmental model study reported that HeyL suppresses *myomaker* expression by binding to the promoter region of *myomaker* [63]. Therefore, one of the reasons that MuSCs express HeyL in hypertrophic muscle may be to avoid untimely fusion and allow their proliferation by suppressing *myomaker* expression. *Myomaker* expression and *HeyL* suppression are necessary for the fusion of MuSCs with myofibers, indicating that the expression of these genes is tightly regulated in proliferating MuSCs during muscle hypertrophy.

### Differences in myonuclear position

The nuclei of regenerating myofibers are positioned in the center of the cytoplasm, which is a signature of regenerated muscle (Fig. 1). Of note, Pawlikowski et al. demonstrated that during middle-late stage of muscle regeneration, a small portion of MuSC-derived nuclei is located in the peripheral position [19]. In mice, the nuclei remain centrally located for at least a few months (personal observation shared by other colleagues).

In contrast, myonuclei derived from MuSCs observed in hypertrophic muscles are located in the peripheral position (Fig. 2), as summarized in our previous review [28]. Many exciting research topics remain to be addressed, such as whether functional differences occur between the central and peripheral nuclei and whether the structure of the nucleus, including the nuclear membrane, or the transcriptional activity/efficiency of the nucleus is the same between central and peripheral nuclei. Moreover, the difference between MuSC-derived and original myonuclei in overloaded muscles must be better understood. Recently, Murach et al. used a loaded spontaneous exercise model and myonuclei-labeling technique to compare the methylation patterns in the promoter region of DNA of resident myonuclei (Mn) present before exercise and the sum of MuSC-derived myonuclei (SC-Mn) and resident myonuclei (Mn+SC-Mn) [64]. The results showed that the Mn+SC-Mn group had a lower methylation state in the region encoding ribosomes, which are necessary for protein synthesis [64]. Although purified SC-Mn must be further investigated to obtain conclusive results, the above findings suggest that SC-Mn are transcriptionally active compared to the original Mn and play a central role in protein synthesis in myofibers. Further characterization of SC-Mn will provide insights on myonuclear function and plasticity, which will lead to the development of therapeutic approaches for muscular atrophy.

### Differences in the number and location of inflammatory cells

Large numbers of neutrophils and macrophages infiltrate muscle tissue during regeneration [24, 65]. During this process, macrophages pass through the basal lamina as macrophages are required to clean the debris of dead myofibers (Fig. 1). Thus, macrophages can directly contact MuSCs in regenerating muscle.

**Fig. 3:**
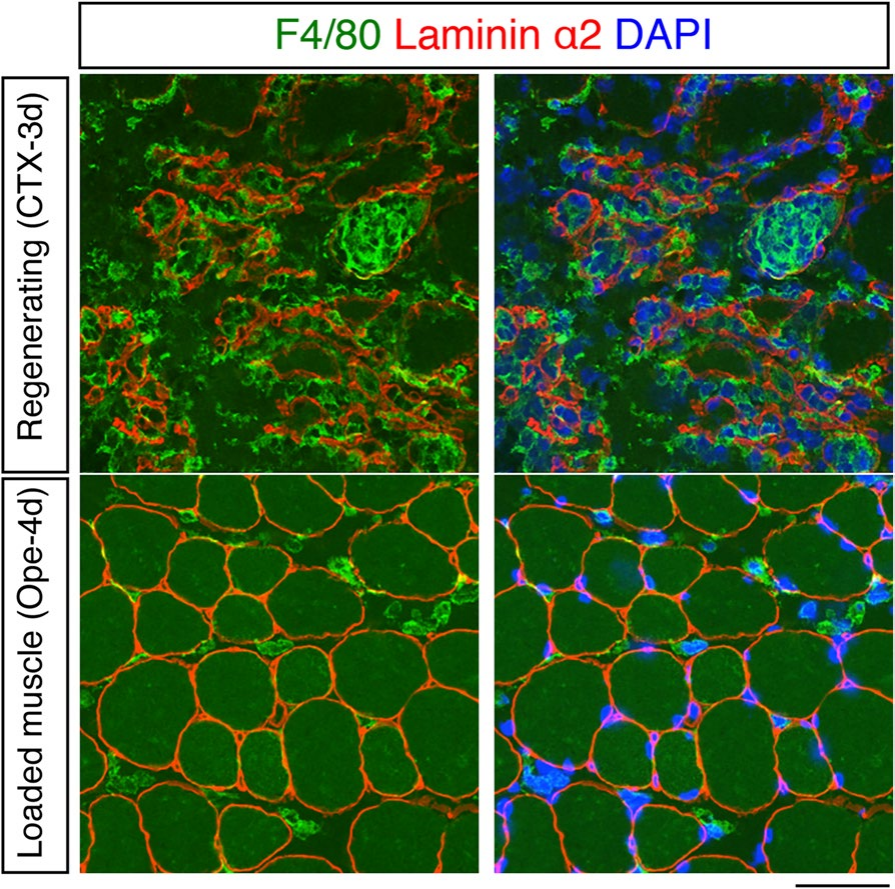
Macrophage infiltration. Immunostaining of F4/80 (green) and laminin α2 (red) in regenerating muscle (upper; from 3 days after cardiotoxin (CTX) injection) and loaded muscle (lower; from 4 days after tenotomy (Ope)). Nuclei were counterstained with DAPI (blue). Scale bar, 50 μm

Compared to the roles of macrophages in regenerating muscle, macrophage functions in loaded muscle are limited, although recent studies have revealed the roles of macrophages in mechanically loaded muscle at the molecular level [10]. Peck et al. reported that macrophages promote extracellular matrix (ECM) remodeling by secreting matrix metallopeptidase 14 (Mmp14) [13], and their data suggest that leukemia inhibitory factor (LIF) from myofibers stimulates Mmp14 expression in macrophages. Noviello et al. demonstrated that macrophages are essential for muscle hypertrophy because macrophage depletion by clodronate liposomes inhibited myofiber growth [10]. The authors also revealed that RhoA signaling in loaded myofibers induces the expression of the chemokine Ccl3/Cx3cl1, which recruits macrophages into the loaded muscle. Overall, although the physiological roles of myofiber-derived factors on macrophages have not been thoroughly examined, the above two studies propose that a myofiber–macrophage axis occurs in muscle hypertrophy. In our unpublished study, macrophage infiltration was observed in overloaded muscle, although the increase in the number of macrophages was low compared with that during muscle regeneration. In addition, in overloaded muscles, macrophages do not need to pass through the basal lamina because myofibers are not damaged. Moreover, macrophage infiltration of the basal lamina has not been observed in the overloaded model except in rare injured areas (Fig. 3). In summary, during muscle hypertrophy, direct contact does not occur between macrophages and MuSCs unless myofibers die. Thus, macrophage-derived factors involved in muscle hypertrophy must be identified to further elucidate the cellular interplay involved in muscle hypertrophy. Interactome analyses of high-quality single-cell RNA-seq data or spatial omics data will fill these gaps and lead to more in-depth understanding of the muscle hypertrophy process.

### Future perspectives

Muscular dystrophy is a representative muscular disorder, and investigations have contributed insights on muscle biology, including the sarcolemma structure, muscle regeneration, and MuSC [66–68]. Muscular atrophy, including sarcopenia, has recently attracted research attention based on the need for new therapeutic approaches for these diseases. Compared with muscular dystrophy, muscle atrophy does not cause severe muscle damage. Briefly, in patients with muscular atrophy, promoting muscle regeneration is not considered a viable therapeutic strategy. Considering the characteristics of muscle atrophy, increasing myofiber size and function are the most promising therapeutic strategies. The myonuclear number has been reported to reflect the myofiber size in both humans and mice [69]. MuSCs are critical for increased myonuclei and myofiber growth [70, 71]; therefore, MuSC-induced increases or improvements in the function of myonuclei in atrophic muscle might contribute to therapeutic approaches. To implement this strategy, the mechanisms regulating MuSC proliferation and differentiation in non-damaged muscles must be better understood.

## Conclusions

Similarities are observed between the regeneration and developmental processes of several tissues, including skeletal muscle. Intriguingly, the “growth” observed in overloaded muscle resembles developmental myogenesis, particularly during postnatal development. Some MuSCs that actively proliferate during postnatal development express weak or undetectable MyoD proteins [72]. Comparable Notch activity likely occurs in postnatal and adult MuSCs because drastic changes in the expression of Notch target genes have not been detected between postnatal and adult MuSCs [73]. In addition, during postnatal development, MuSCs proliferate between the basal lamina and myofibers and inflammatory cells are not observed. Furthermore, myonuclei derived from MuSCs are peripheral during postnatal development. Based on this evidence, we conclude that the dynamics of MuSCs in overloaded muscle could be considered an intermediate cellular event between muscle regeneration and postnatal developmental myogenesis. Further investigations of MuSCs in overloaded muscle, which is distinct from regeneration and developmental muscle models, will provide new insights on MuSC biology and therapeutic approaches for muscle atrophy.

## Data Availability

Not applicable

## Abbreviations

*Col5a1*: Collagen type V alpha 1 chain
CTX: Cardiotoxin
ECM: Extracellular matrix
Foxo: Forkhead box O
GDF: Growth differentiation factor
Igf1: Insulin-like growth factor 1
*Hey1*: Hairy/enhancer-of-split related with YRPW motif protein 1
*HeyL*: Hairy/enhancer-of-split related with YRPW motif-like protein
LIF: Leukemia inhibitory factor
Mmp14: Matrix metallopeptidase 14
mTOR: Mammalian target of rapamycin
MuSCs: Muscle satellite cells
MyoD: Myoblast determination protein 1
*Pdgfrα*: Platelet-derived growth factor receptor alpha
Thbs1: Thrombospondin-1
WISP1: WNT1 inducible signaling pathway protein 1
